# Long-Term Outcomes in Patients Managed with the Endurant^TM^ Endograft under Elective Setting

**DOI:** 10.3390/jcm13185601

**Published:** 2024-09-21

**Authors:** Konstantinos Spanos, Petroula Nana, George Volakakis, George Kouvelos, Konstantinos Dakis, Christos Karathanos, Eleni Arnaoutoglou, Miltiadis Matsagkas, Athanasios Giannoukas

**Affiliations:** 1 Department of Vascular Surgery, Larissa University Hospital, Faculty of Medicine, University of Thessaly, 41110 Larissa, Greece; geo-vol@hotmail.com (G.V.); geokouv@gmail.com (G.K.); kostasdakis1994@gmail.com (K.D.); christos2001@hotmail.com (C.K.); milmats@gmail.com (M.M.); agiannoukas@hotmail.com (A.G.); 2 German Aortic Centre, Department of Vascular Medicine, University Heart and Vascular Centre UKE Hamburg, 20246 Hamburg, Germany; petr.nana7@hotmail.com; 3 Department of Anesthesiology, Larissa University Hospital, Faculty of Medicine, University of Thessaly, 41110 Larissa, Greece; earnaout@gmail.com

**Keywords:** aneurysm, Endurant, endovascular repair, long-term outcomes, mortality, reintervention

## Abstract

**Background/Objectives**: Device selection during endovascular aneurysm repair (EVAR) for abdominal aortic aneurysms (AAAs) remains an important issue for ensuring endograft durability. This study evaluated the early and follow-up outcomes of elective EVAR with the Endurant platform. **Methods**: A single-center retrospective analysis was conducted including consecutive elective EVAR procedures with the Endurant II/IIs (2008 to 2024) device. Primary outcomes were technical success, mortality and major complications at 30 days. Survival, endoleak I/III and freedom from reintervention were secondary outcomes. Cox proportional hazards models were employed for risk-adjusted follow-up outcomes. **Results**: In total, 361 patients were included (72.7 ± 7 years; 96% males; mean AAA diameter 62 ± 14 mm); 92% received a bifurcated device, and 89% conformed to the instructions for use. Technical success was 99.7%. Intra-operative adjunctive procedures included 4.4% proximal cuffs and 1.7% endoanchors. The thirty-day mortality rate was 0.6%, and the major complication rate was 4.1%. Survival was 81% (SE 4.8%), 72% (SE 6.4%) and 52% (SE 9.2%) at 4, 6 and 8 years, with aneurysm-related mortality at 1.7%. Freedom from endoleak Ia was 76% (SE 7.3%) at 6 years, freedom from endoleak Ib was 79% (SE 7.4%) at 7 years and freedom from endoleak III was 94% (SE 3.7%) at 5 years. Freedom from reintervention was 71% (SE 6.1%) and 55% (SE 7.9%) at 5 and 7 years, respectively. No device-related co-factor affected long-term outcomes. **Conclusions**: Endurant II/IIs endograft is a safe and effective EVAR solution with excellent early outcomes and low long-term aneurysm-related mortality. The need for reintervention in the long term affected less than 50% of cases.

## 1. Introduction

Endovascular aneurysm repair (EVAR) has become the standard treatment for abdominal aortic aneurysms (AAAs). Thus, for most patients with suitable anatomy and reasonable life expectancy, endovascular repair should be considered as the preferred treatment modality for elective abdominal aortic aneurysm repair according to the latest (2024) guidelines by the European Society of Vascular Surgery (ESVS; Class IIa, Level of Evidence B) [1]. However, long-term durability remains still an issue, with EVAR showing higher reintervention rates and aneurysm-related mortality compared to open surgical repair [2,3,4]. For this purpose, the recent ESVS guidelines presented a new recommendation highlighting that device selection should be considered based on aorto-iliac anatomy and the availability of unbiased long-term durability data (Class IIa, Level of Evidence C) [1].

The Endurant stent graft (Medtronic Endovascular, Santa Rosa, Calif) has been widely used since receiving the CE mark in Europe in 2008. Robust data have been available from The Endurant Stent Graft Natural Selection Global Post-market Registry (ENGAGE) which is a global, prospective multicenter registry evaluating the Endurant stent graft system (Medtronic, Santa Rosa, CA, USA) and represents the largest registry for any single EVAR stent graft [5]. The 10-year data of the ENGAGE registry showed good long-term outcomes with a 94.7% freedom from aneurysm-related mortality (ARM) and 64.1% sac regression at 10 years [6]. Real-world data, out of registries, showed encouraging early and follow-up outcomes using the Endurant device for elective AAA management [7].

Thus, the current study aimed to evaluate real-world early and long-term outcomes of the Endurant stent graft platform in elective AAA treatment in a tertiary reference center.

## 2. Materials and Methods

### 2.1. Study Design

A single-center retrospective analysis was conducted including consecutive patients managed electively with Endurant II/IIs for infra-renal abdominal aortic aneurysms (AAAs) between 2008 and 2024, and in accordance with the STrengthening the Reporting of OBservational studies in Epidemiology (STROBE) guidelines [8]. This study complied with the Declaration of Helsinki, and ethical approval was waived, given the retrospective design and use of anonymized data.

### 2.2. Patient Population

All patients were treated using the Endurant II/IIs endograft (Medtronic, Santa Rosa, CA, USA), bifurcated or aorto-uni-iliac (AUI), under an elective setting for infra-renal AAAs. The indications to treat degenerative AAAs relied on the available aortic guidelines at the time of repair [1,9,10]. Sizing and planning were performed by experienced vascular surgeons, based on a recent (within 3–6 months) pre-operative computed tomography angiography (CTA), using dedicated software (3Mensio, Medical Imaging B.V., Bilthoven, the Netherlands). All interventions were performed in an adequately equipped operating room, using a moveable radiolucent surgical table (2008–2019: Maquet, Getinge Group, Rastatt, Germany; 2019–2024: Stille Surgical, Inc., Torshälla, Sweden) and a mobile digital angiographic system (2008–2019: Philips BV Endura, Philips Medical Systems, Release 2.2.3, Amsterdam, the Netherlands; 2019–2024: Ziehm Vision RFD Hybrid Edition, Ziehm Imaging, Nuremberg, GE).

Procedures were performed after appropriate anesthesiologic evaluation. The pre-operative work-up included cardiologic and pulmonary evaluation, with transthoracic echocardiography and spirometry, respectively. Further evaluation was decided depending on the patients’ findings. According to the pre-operative assessment, patients were preferably managed using general anesthesia to provide optimal endograft deployment with elimination of respiratory movements. Epidural anesthesia and local anesthesia with monitored anesthesia care were used in the remaining cases.

Femoral access was performed using bilateral cut-down in all cases. The bifurcated Endurant device was used in most cases. Usually, a right access was preferred for main device advancement, and only when inadequate right access (<7 mm diameter, severe stenosis >70% or occlusion) was present, a left access was used. After an initial angiography for the identification of the lowest renal artery, the main device was deployed, and the contralateral limb was catheterized in standard fashion; this was followed by the deployment of both iliac limbs, after the angiographic identification of both internal iliac artery (IIA) origins. In the case of a large common iliac artery (18–24 mm), either the bell bottom technique was used or an IIA overstenting (>24 mm) and landing to the external iliac artery (EIA) was performed. The AUI device was applied in some cases, depending on distal landing zones, surgeon’s preference and availability of the device, especially during the earlier experience [11]. For patients managed with the AUI device, an iliac occluder was deployed to the contralateral iliac artery, and a femoral–femoral bypass was performed using a synthetic 8 mm graft (W. L. Gore & Associates, Flagstaff, AZ, USA). Uncomplicated cases were post-operatively transferred to the ward for at least 24 h.

If an uneventful post-operative course occurred, the patients were discharged the 2nd or 3rd post-operative day, with a re-evaluation at 1, 6, 12 months and yearly thereafter. The 1- and 12-month evaluations were performed using a CTA, depending on the patient’s renal function [12]. Duplex ultrasonography was preferred for the 6-month evaluation and the yearly follow-up after 12 months. The imaging protocol could present alterations depending on imaging findings, the presence of endoleak and the patient’s symptoms.

### 2.3. Exclusion Criteria

Patients managed with the Endurant platform or components (e.g., limbs or cuffs) combined with other devices (e.g., main aortic grafts, iliac branched devices) were excluded. Adjunctive procedures decided according to the intra-operative imaging, such as the use of cuffs, endoanchors or a kissing stent of the iliac limbs were not considered as a criterion for exclusion. Patients scheduled for an endovascular repair with the Endurant endograft, needing the inclusion of visceral vessels, using the parallel graft technique, were omitted. Patients managed under urgent circumstances including ruptured or symptomatic AAAs were also excluded.

### 2.4. Device and Technical Details

The bifurcated Endurant II/IIs device was used in all cases. Regarding the Endurant characteristics, the stents are M-shaped, made of nitinol and sewn to a polyester fabric graft while proximal sealing is secured with active suprarenal fixation, having barbs in the suprarenal stent. According to the device’s instructions for use (IFU), a neck of 15 mm associated with an infra-renal angle <75 and non-severe thrombus or atheroma is needed. In case the neck angle is <60, a neck length of 10 mm is also considered adequate for Endurant’s deployment. The bifurcated body requires one or two iliac extensions, and a common iliac artery up to 24 mm is considered manageable with the Endurant’s larger distal limb of 28 mm. The delivery system is sheath-less, with a profile of up to 20 Fr for the main body, meaning that an iliac diameter of at least 7 mm is needed for safe main device advancement.

### 2.5. Data Collection

A dedicated database was created for the collection of pre-, intra- and post-operative data, which were inserted after pseudonymization by an experienced study nurse and vascular surgeons. Clinical and imaging data of any follow-up evaluation were added to the same database. Patients who did not attend the scheduled follow-up with evaluation at least once per two years, after the index procedure, were considered as lost to follow-up.

### 2.6. Definitions

The infra-renal diameter was considered the outer–outer wall diameter of the aorta just below the inferior main renal artery [13]. Neck thrombus and calcification were expressed as percentages of the aortic circumference, with 0–25% considered as mild, 25–50% as moderate and >50% as severe [14]. The presence of calcification/thrombus in >50% of vessel length was reported as severe iliac thrombus or calcification [15]. A diameter of the CIA over 18 mm was used to identify an aneurysmal CIA [16]. Technical success and major systemic complications (MSCs) were reported according to the Society of Vascular Surgery Reporting Standards for endovascular aortic aneurysm repair [17]. According to the Society of Vascular Surgery guidelines, the device oversizing at the level of the infra-renal neck should be 10–20% [18,19]. The oversizing was calculated according to the average neck diameter throughout the intended sealing zone [18,19].

### 2.7. Outcomes

Primary outcomes were the technical success, mortality and MSC rates at 30 days. Survival, freedom from endoleak types I and IIIa and freedom from reintervention during long-term follow-up were considered secondary outcomes.

### 2.8. Statistical Analysis

Normally distributed continuous data were reported as mean–standard deviation, and non-normally distributed as median values with range and IQR. Categorical data were expressed as absolute numbers and percentages. The chi-square test was used for categorical data comparison. Independent two-sample t tests were used for normally distributed continuous variables, and the Mann–Whitney U test for non-normally distributed continuous and ordinal variables. Due to the lack of adverse events in the early post-operative period, factors affecting outcomes could not be reliably controlled using regression analysis according to the rule of thumb. Kaplan–Meier estimates were performed to assess follow-up outcomes. To determine risk-adjusted follow-up outcomes, Cox proportional hazards models were employed, adjusting for known variables, as they were detected previously in the literature: neck diameter and length, conformity to IFU and oversizing. No correction for multiple hypothesis testing was applied. The sample size was allowed to vary based on the analysis, and no imputation of missing data was performed as missing data were infrequent (<5%) for both categorical and continuous variables. The *p* value was considered significant when it was <0.05 (two-tailed hypothesis). Statistical analysis was performed by SPSS 29.0 for Windows software (IBM Corp, Armonk, NY, USA)

## 3. Results

### 3.1. Patients’ Cohort

From 2008 to 2024, 361 patients were treated electively with standard EVAR using the Medtronic II/IIs stent graft in a single tertiary center. The mean age was 72.7 ± 7 (52–92) years, and 96% (347/361) were males. The mean AAA diameter was 62 ± 14 mm. Patients’ comorbidities are presented in Table 1.

Most patients were treated with a bifurcated device (92%; 332/361), and 89% (324/361) of the patients were treated according to the IFUs of the device. Pre-operative anatomic characteristics of the aorta and the iliac arteries are shown in Table 2.

### 3.2. Intra-Operative Findings

Epidural or local anesthesia was chosen for 53% (193/361) of patients, while 47% of the patients (168/361) were treated under general anesthesia. The technical success rate was 99.7% (360/361); in one patient, an open conversion was needed. In 42 (11.6%) patients (in three, bilaterally), at least one internal iliac artery was overstented, and landing to the EIA was chosen. Intra-operative unscheduled adjunctive procedures included 15 patients with proximal cuff deployment and 6 patients with endoanchor treatment; all endoanchors were applied in cases with a type Ia endoleak shown in the completion angiography, while the use of cuffs was related to intra-operative caudal device migration, with or without the presence of endoleak. The final decision was at the discretion of the treating physician. The mean operative time was 135 ± 56 min, and the mean contrast volume was 108 ± 101 mL.

### 3.3. Early Clinical Outcomes

The 30-day mortality was 0.6% (2/361). One patient developed multiorgan failure, and one patient died from a cardiac cause. Major complications were recorded in 15 patients (4.1%): 2 (0.5%) patients suffered a myocardial infarction, 5 (1.3%) needed access reintervention, 5 (1.3%) presented with acute limb ischemia and 3 (0.8%) with acute kidney injury (AKI) needing dialysis. The median hospital stay was 4 (IQR 1) days. In 14% of patients, a blood transfusion was needed during hospitalization.

### 3.4. Follow-Up Outcomes

**Figure 1:**
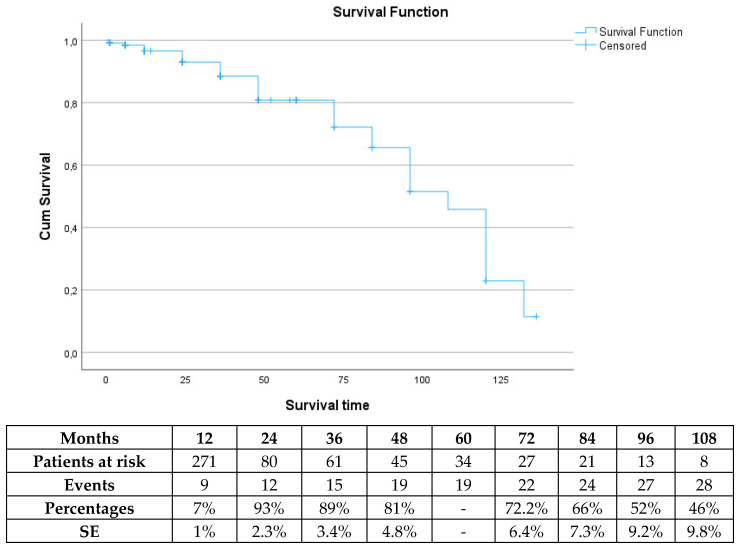
Survival during the follow-up period in elective cases managed with the Endurant device. SE: standard error.

The mean follow-up was 20.9 ± 25 months, with 30.4% being lost to follow-up. The survival rate was 93% (SE 2.3%), 81% (SE 4.8%), 72% (SE 6.4%) and 52% (SE 9.2%) at 2, 4, 6 and 8 years, respectively (Figure 1). In total, 32 patients died during follow-up. Six patients presented with ruptured EVAR; four underwent urgent open conversion (two of them survived), and two died without intervention. After Cox regression analysis was performed, defining neck diameter [hazard ratio (HR) 1.1, 95% CI 0.9–1.3, *p* = 0.08] and IFU (HR 0.2, 95% CI 0.03–1.2, *p* = 0.08) as co-factors, neither co-factor was shown to affect the long-term survival.

**Figure 2:**
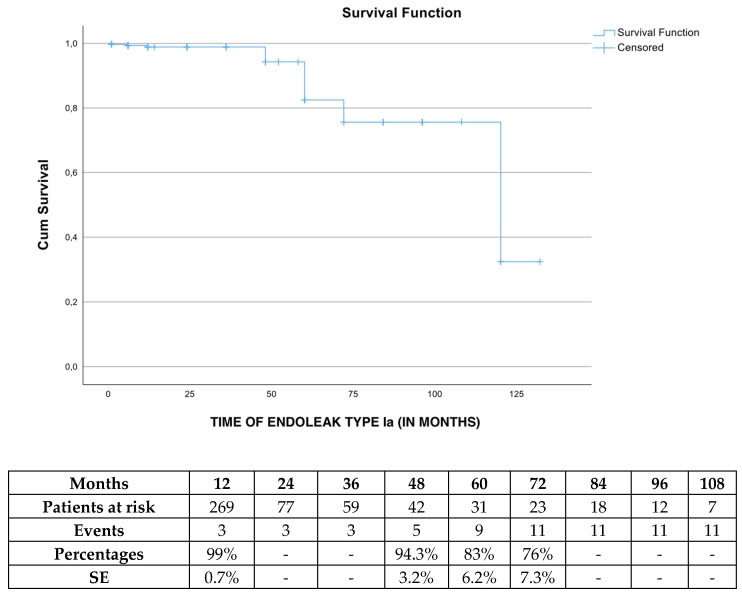
The estimated freedom from endoleak type Ia during follow-up in elective cases managed with the Endurant device. SE: standard error.

The freedom from endoleak Ia rate was 99% (SE 0.7%), 94.3% (SE 3.2%) and 76% (SE 7.3%) at 1, 4 and 6 years, respectively (Figure 2). After Cox regression analysis was performed, defining oversizing rate (HR 3.15, 95% CI 0.4–23.5, *p* = 0.26), neck length (HR 1.1, 95% CI 0.19–6.4, *p* = 0.9) and neck diameter (HR 1.12, 95% CI 0.9–1.39, *p* = 0.28) as co-factors, none of the co-factors was shown to affect endoleak Ia.

**Figure 3:**
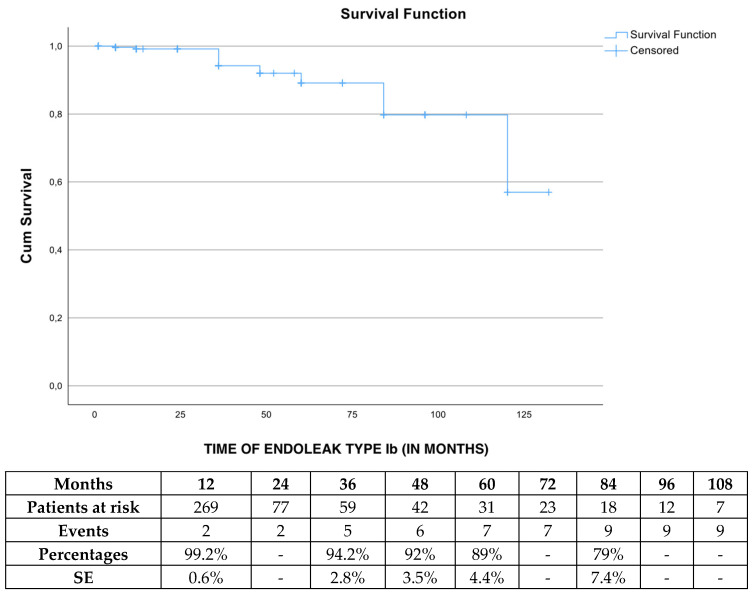
The estimated freedom from endoleak Ib rate during follow-up period in elective cases managed with the Endurant device. SE: standard error.

The estimated freedom from endoleak type Ib was 99.2% (SE 0.6%), 92% (SE 3.5%) and 79% (SE 7.4%) at 1, 4 and 7 years, respectively (Figure 3), with no event recorded during the 1st-month follow-up. After Cox regression analysis was performed, defining IFU (HR 0.7, 95% CI 0.04–11,7 *p* = 0.07) as a co-factor, no impact on endoleak type Ib was detected. No patient with CIA diameter >18 mm presented an endoleak type Ib.

**Figure 4:**
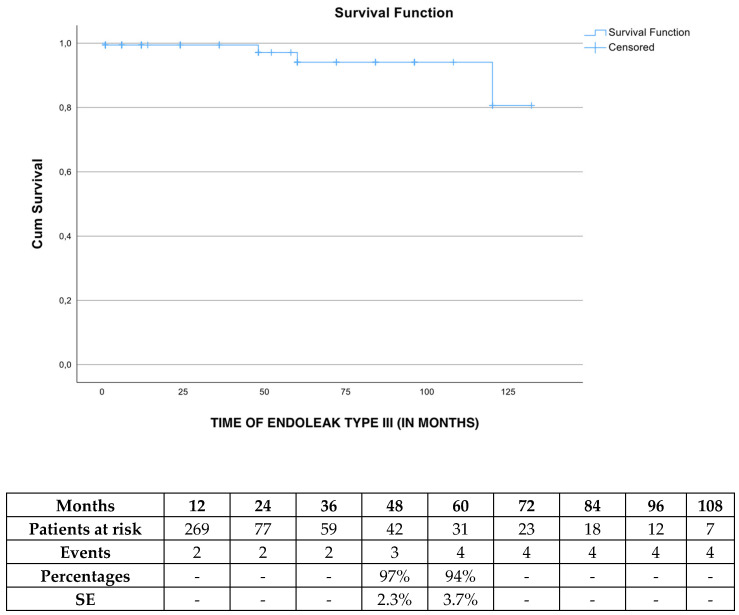
The estimated freedom from endoleak type III rate during follow-up in elective cases managed with the Endurant device. SE: standard error.

The freedom from endoleak type III was 97% (SE 2.3%) and 94% (SE 3.7%) at 4 and 5 years, respectively (Figure 4), while one more patient presented with an ET III at 120 months of follow-up. After Cox regression analysis was performed, defining IFU (HR 25.1 95% CI 0.00–22489 *p* = 0.58) as a co-factor, no impact was detected.

**Figure 5:**
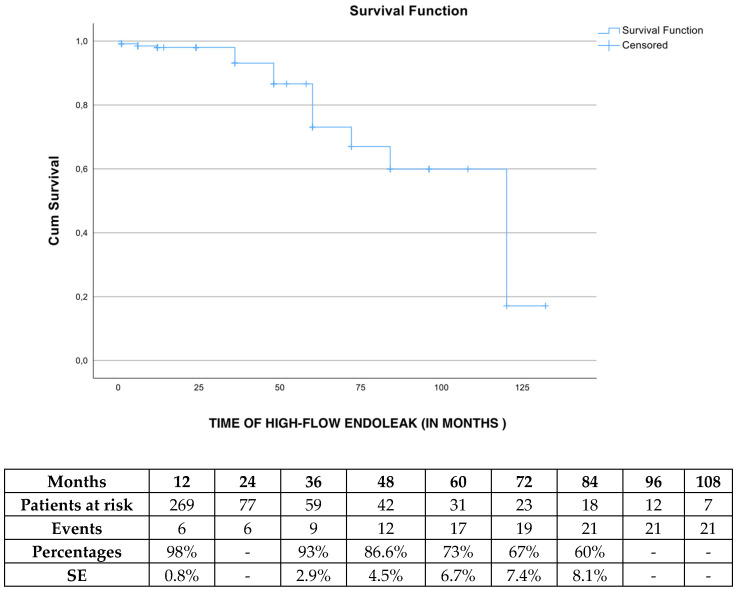
The estimated freedom from any high-flow endoleak (type Ia, Ib and III) rate during follow-up in patients managed electively with the Endurant device. SE: standard error.

The freedom from any high-flow endoleak (type Ia, Ib and III) was 98% (SE 0.8%), 93% (SE 2.9%), 73% (SE 6.7%) and 60% (SE 8.1%) at 1, 3, 5 and 7 years, respectively (Figure 5). Regarding endoleak type II, the estimated freedom from endoleak type II rate was 75% (SE 3.2%) at 36 months of follow-up.

**Figure 6:**
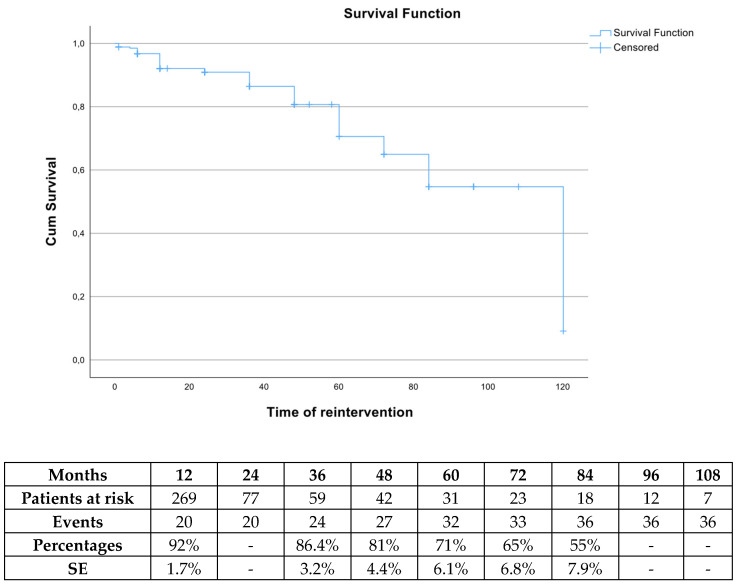
The estimated freedom from any reintervention rate during follow-up in patients managed electively with the Endurant device. SE: standard error.

Freedom from reintervention rate was 92% (SE 1.7%), 86.4% (SE 3.2%), 71% (SE 6.1%) and 55% (SE 7.9%) at 1, 3, 5 and 7 years, respectively (Figure 6). After Cox regression analysis was performed, defining Endoleak type II as a potential parameter for reintervention, no significance was detected (HR 1.53 95% CI 0.7–3.35 *p* = 0.21). In total, 41 patients received 54 reinterventions, as depicted in Table 3. In addition to high-flow endoleak management, one patient was managed for graft infection and one for an aortoenteric fistula with severe bleeding, while two patients presented with total graft thrombosis. Iliac limb occlusion affected eleven patients, while three patients presented iliac limb stenosis and were treated preventively.

## 4. Discussion

The Endurant endograft has been related to promising long-term outcomes at 8 years of follow-up, with freedom from aneurysm rupture at 95% and freedom from endoleak I/III at 87.3% [7]. However, the published literature reporting data over five years of follow-up is quite limited [6,7]. The current analysis including real-world experience data showed a 72% and 52% survival at 6 and 8 years of follow-up, respectively, with very low aneurysm-related mortality, freedom from high-flow endoleaks at 60% at 7 years and freedom from reintervention at 55% at 7 years. The early outcomes, including technical success, mortality and major complications, once again confirmed the safety of the Endurant endograft, as also previously described in the ENGAGE registry [20]. Early EVAR safety has been previously confirmed and allowed it to be established as the first-line treatment in patients with appropriate anatomy [1]. However, long-term endoleak formation and the associated high rate of reintervention set still the durability of the technique in disguise [1,21].

The recent guidelines of the ESVS suggest the use of EVAR, as first-line treatment, in appropriately selected patients while they highlight the role of adequate device selection relying not only on the patient’s anatomy but further on the long-term durability outcomes of the devices, as reported in the literature [1]. In addition, the recent guidelines advise the use of the devices according to the specific IFU, as defined by each manufacturer [1]. However, the previously published experience is rather conflicting regarding the impact of IFUs in real-world studies [22,23]. In our analysis, IFU was not detected as a predictor of adverse events after it was included as a co-factor in the Cox regression analyses [24].

Regarding the early outcomes, including technical success, mortality and major complications, Endurant performed with very high safety, as detected by the associated rates and the sporadic adverse events. The early and midterm outcomes of the EAGLE registry confirmed that, even when applied in challenging anatomies, Endurant can provide a high technical success rate and low aneurysm-related mortality [25]. As early findings seem to affect long-term outcomes, in terms of survival, need for reintervention and risk of aneurysm rupture, initial technical success seems of major importance [26]. In this analysis, 5.8% of patients needed an additional intra-operative maneuver to secure the proximal sealing zone. Adjunctive procedures, such as the use of aortic cuffs or endoanchors, when needed, should be performed to increase the stability of the device in the proximal sealing zone [27].

The survival was estimated at 72% and 52% at 6 and 8 years, respectively, in the current cohort. However, only six events of aneurysm-related mortality were detected, showing that the long-term survival may be affected by other underlying diseases and parameters. Previous meta-analytic data seem to confirm this steep decrease in survival when passing from the mid-term to long-term follow-up and relate this finding to the aneurysm-related mortality, a pattern that does not seem to be confirmed in the current analysis [21]. Patient age and underlying comorbidities seem to affect significantly the long-term outcomes of EVAR in an elective setting, with older and multimorbid patients being at higher mortality risk [28]. In our cohort, coronary artery disease was the third most common comorbidity, affecting 40% of patients and potentially affecting long-term survival [29]. In addition, the use of Endurant out of the IFU and in large necks did not affect outcomes, despite previous studies showing that both factors may play a significant role in survival [22,30].

High-flow endoleaks were detected in almost 30% of patients at 7 years of follow-up, with type Ia being detected in 25% of them during the same period. Supra-renal fixation may be related to proximal aortic dilation during the mid-term follow-up and potentially lead to endoleak formation during the long-term surveillance, highlighting the potential necessity of using endoanchors primarily to prevent neck dilatation after EVAR. [12,31,32]. Available Endurant long-term data showed similar to the current analysis rates of endoleak type I/III at 8 and 10 years of follow-up, while the application of Endurant out of the IFU has been detected as an independent predictor for high-flow endoleaks [7,21]. Type II endoleaks were not further investigated in this study, due to their multifactorial nature [1]. However, previous studies showed that device material may affect endoleak type II formation up to 2 years of follow-up [33,34]. Anatomic parameters, including large proximal neck and iliac diameters, and application of the device out of the IFU did not provide significance in Cox regression analyses of endoleaks, showing that the device can provide safety even in unfriendly anatomies [22,30].

Regarding reinterventions, less than half of patients needed a secondary intervention during the long-term follow-up. However, it should be noted that most reinterventions were performed in 41 patients; most of them were treated during the 1st year and 50% of them were related to distal sealing or patency complications and were relatively minor ones [7]. The limb occlusion rate has been reported at 5% during the mid-term follow-up, while loss of distal sealing seems to affect almost 20% of patients and has been previously related to dilated distal sealing zones [16,31]. Except for the four open conversions, all complications related to the proximal sealing zone were resolved using endovascular means [21]. Open conversion after failed EVAR has been related to high mortality rates, especially when patients present with ruptured AAAs [35]. On the other hand, endovascular management with fenestrated or branched devices after failed EVAR has been related to 2.4% mortality and high technical success when performed by experienced hands [36]. Especially regarding the secondary endovascular management of a failed Endurant graft, the presence of the wide diameter bare stent, despite challenges, permits the catheterization of the visceral vessels and the completion of the procedure in most cases.

Endurant seems to be a safe solution in the short term according to previous data and the findings of the current analysis [20]. The presence of highly visible markers, the ease of device deployment and the conformity of the device even in extreme proximal angulations make the Endurant an attractive solution. The long-term data follow the literature on general EVAR performance, with a high need for reinterventions during the long-term follow-up [37]. The long-term survival may seem discouraging, but it should be noted that EVAR is still performed in elder patients with high-risk profiles. However, the aneurysm-related mortality remains low, showing that Endurant is a durable and reliable solution for elective AAA management, even when applied out of IFU and in hostile neck anatomies, while further investigations on the use of endoanchors, even in non-hostile necks, may be able to decrease significantly the presence of proximal endoleaks and reinterventions [27,38,39]. The Cox regression analysis failed to confirm any correlation to hostile anatomic factors and rather confirms Endurant’s reliability but at the same time raises questions about the predictability of EVAR failure, in hostile and non-hostile anatomies, and appropriate surveillance protocols [40]. Standardized follow-up imaging, in rational time intervals, and using the principle of as little radiation exposure as possible, is important for the prevention and early detection of complications. Social and economic factors, health system facilities and patient’s needs, though, should be taken into consideration to provide appropriate surveillance.

### Limitations

The retrospective design of the study and the relatively small sample size may have influenced the findings of the analysis. No comparisons were attempted between the Endurant device and other widely used available devices, while patients who were managed with a combination of devices or received Endurant components (e.g., cuffs, iliac limbs, tube grafts) were excluded from the analysis to increase homogeneity. The latest criterion led to the exclusion of patients managed with iliac branched devices for the preservation of the hypogastric artery, as the Endurant platform does not provide such an endovascular solution. A further investigation of the parameters affecting different types of reinterventions and their etiology was not attempted, as such a subgroup analysis would be significantly underpowered, and the outcomes would not be reliable (rule of thumb). On the other hand, patients with AUI were included and may have affected the findings, especially regarding distal landing zone adverse events and reinterventions, while it should be noted that AUI devices mainly represent the earlier experience of our department. Iliac tortuosity was not analyzed, due to a lack of information, but may have affected outcomes in terms of iliac limb adverse events. Percutaneous access was not employed in any Endurant case, due to bureaucratic reasons only. Patients lost to follow-up may have potentially affected our outcomes and decreased the robustness of our follow-up findings. Although neither neck dilation nor IFU affected our findings, this fact should be interpreted cautiously, as the currently available literature is rather conflicting. Endoleak type II was not investigated in detail, as it is considered a multifactorial phenomenon, whose evolution is affected by anatomic, pharmacological and technical factors, and a direct correlation between Endurant’s performance and endoleak type II would be difficult to establish.

## 5. Conclusions

The Endurant II/IIs endograft is a safe and effective elective EVAR solution with excellent early outcomes and very low long-term aneurysm-related mortality. The need for reintervention in the long term affected less than 50% of cases. Type Ia/b endoleaks were encountered in 30% of patients during the long-term follow-up, while type III was less common.

## Figures and Tables

**Table 1:** Patients’ comorbidities (CAD: coronary artery disease; CABG: coronary artery bypass graft; PCI: percutaneous coronary intervention; COPD: chronic obstructive pulmonary disease; CRD: chronic renal disease; eGFR: estimated glomerular filtration rate).

Comorbidity	Number of Patients
Age (years)	72.7 ± 7
Males	347 (96%)
Aneurysm diameter (mm)	62 ± 14
Hypertension	276 (77%)
Dyslipidemia	231 (64%)
Tobacco use	221 (61%)
CAD	148 (41%)
CABG	56 (16%)
PCI	50 (14%)
COPD	94 (26%)
CRD	26 (7%)
Renal dialysis	12 (3%)
Creatinine (mg/dL)	1.1 ± 0.4
eGFR (mL/min)	77.1 ± 6.3
Pre-operative medical treatment	
Acetylsalicylic acid	187 (52%)
Clopidogrel	44 (12%)
Double aantiplatelet	22 (6%)
Anticoagulant	43 (12%)
Statin	202 (56%)

**Table 2:** Pre-operative anatomical characteristics of the aorta and the iliac arteries; AAA: abdominal aortic aneurysm; RCIA: right common iliac artery; REIA: right external iliac artery; LCIA: left common iliac artery; LEIA: left external iliac artery.

Anatomic Aortic Characteristic	Value
Mean AAA Diameter (mm)	62 ± 14
Neck Characteristics	
Mean Diameter (mm)	24.7 ± 5
Mean Length (mm)	24 ± 11
Mean Infra-renal Angulation (°)	32 ± 20
Mean Supra-renal Angulation (°)	31.5 ± 19
No Neck Calcification	177
Mild Neck Calcification	107
Moderate Neck Calcification	20
Severe Neck Calcification	9
No Neck Thrombus	150
Mild Neck Thrombus	97
Moderate Neck Thrombus	49
Severe Neck Thrombus	6
Iliac Artery Characteristics	
Mean RCIA Diameter (mm)	16 ± 8
Mean RCIA Angulation (°)	40 ± 22
No RCIA Calcification	79
Mild RCIA Calcification	149
Moderate RCIA Calcification	94
Severe RCIA Calcification	39
Mean REIA Diameter (mm)	9 ± 1.6
Mean LCIA Diameter (mm)	15 ± 5
Mean LCIA Angulation (°)	45 ± 25
No LCIA Calcification	79
Mild LCIA Calcification	150
Moderate LCIA Calcification	83
Severe LCIA Calcification	49
Mean LEIA Diameter (mm)	9.1 ± 1.5

**Table 3:** Time and type of reinterventions that patients received during the follow-up period. N: number; fem-fem: femoro-femoral; ax-fem: axillo-femoral; AUI: aorto-uni-iliac; PG-EVAR: parallel graft endovascular aortic aneurysm repair; BEVAR: branched endovascular aortic aneurysm repair.

Time in Months	N of Patients	Open Conversion	Aortic Cuff	Iliac Extension	Iliac Relining	Femoral-Femoral Bypass	Axillo-Femoral Bypass	AUI	PG-EVAR	BEVAR	Type II Endoleak Embolization
1	4	0	0	1	1	2	0	0	0	0	0
12	16	0	2	1	3	4	3	1	1	0	1
24	0	0	0	0	0	0	0	0	0	0	0
36	4	0	0	1	0	1	1	0	0	0	1
48	3	1	1	1	0	1	0	0	0	1	2
60	5	1	3	1	1	0	0	0	1	0	0
72	1	0	1	0	0	0	0	0	0	0	0
84	3	0	2	2	1	0	0	0	0	0	0
120	5	1	1	1	2	1	0	1	1	1	1
Total	41	3	10	8	8	9	4	2	3	2	5

## Data Availability

Data are available upon reasonable request from the corresponding author.
